# #GERIATRICS: AN ANALYSIS OF THE IMPACT OF THE GERIATRICS TWITTER NETWORK

**DOI:** 10.1093/geroni/igz038.1223

**Published:** 2019-11-08

**Authors:** Varun Ayyaswami, Arpan Prabhu, Steven Gambert

**Affiliations:** 1 University of Maryland School of Medicine, Baltimore, Maryland, United States; 2 University of Pittsburgh School of Medicine, Pittsburgh, Pennsylvania, United States

## Abstract

Twitter connects an international community of healthcare stakeholders, potentially augmenting access to information related to geriatric medicine. The purpose of this study is to analyze the geriatric medicine Twitter network, and we hypothesize this community has substantially grown in the last six years. We analyzed all publicly available tweets including the hashtag #geriatrics from January 1, 2013-January 1, 2019 using Symplur Signals, a health care social media analytics platform. We evaluated #geriatrics metrics over time related to activity, content analysis, user characteristics, engagement, and network analysis. A total of 159,008 tweets (containing 42.8% re-tweets) with the hashtag #geriatrics were written by 29,443 users, resulting in 393.6 million impressions. The number of tweets increased from 9,705 in 2013 to 39,151 in 2018 (32.2% compound annual growth); users increased from 3,366 in 2013 to 29,443 in 2018 (55.3% compound annual growth). Users were primarily found in the United States, United Kingdom, and Canada. The most commonly trending topic from 2013-2015 and from 2016-2018 was ‘older adults’. The top hashtags included in tweets with #geriatrics were #job, #aging, and #hpm (hospice and palliative medicine). Network analysis showed central hubs to be medical journals, provider organizations, individual physicians, and individual advocates. The top 150 influencers consisted primarily of physicians (49.1%), advocate/support organizations (13.8%), and media organizations (6.3%). The use of Twitter to promote geriatric medicine using #geriatrics has matured into an international digital community of interest. Future studies should examine hashtags related to age prevalent illnesses and hashtags likely to be used by patients.

